# Delayed Treatment of Retroclival Meningioma in a 70-year-old Female

**DOI:** 10.7759/cureus.6579

**Published:** 2020-01-06

**Authors:** Vivian S Tan, Lara Best

**Affiliations:** 1 Department of Radiation Oncology, Dalhousie University, Halifax, CAN

**Keywords:** meningioma, radiation oncology, delayed treatment

## Abstract

We report a case of a 70-year-old female who presented with a four-year history of progressive headaches in the occipital area. MRI revealed a right inferior clival meningioma. Treatment was delayed for over a year due to multiple referrals resulting in the development of new symptoms including decreased balance, generalized weakness, and difficulty swallowing.

## Introduction

Meningiomas account for approximately one-third of all tumors in the central nervous system [1]. They are slow-growing tumors that arise from the dura and are often benign and asymptomatic. Asymptomatic meningiomas typically remain the same size or grow slowly over prolonged periods [2]. However, symptoms can occur depending on the location of the mass. For small, stable, and asymptomatic meningiomas, active surveillance is often recommended [3]. Active treatment for meningiomas may involve complete surgical resection, subtotal resection with or without radiation therapy, or radiation therapy alone. Choice of treatment depends on the overall patient condition, location of the mass in relation to critical brain structures, and histopathologic characteristics of the tumor. The aim of the case report is to demonstrate the progression of a symptomatic meningioma with delayed treatment.

## Case presentation

**Figure 1:**
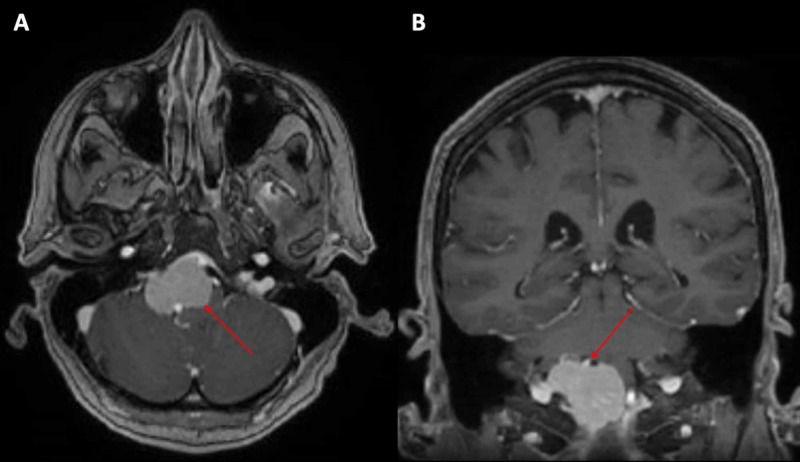
(A) Axial and (B) coronal views of magnetic resonance imaging of the brain at the time of diagnosis revealing right inferior clival meningioma.

A 70-year-old female first presented with a four-year history of progressive headaches in the occipital area. She described her headaches as a pressure sensation that worsened with a cough or a sneeze. The headaches started to increase, occurring daily over the last two years. Due to these findings, she underwent an MRI of the brain which showed a right inferior clival meningioma extending across the top of the jugular tubercle and projecting into the anterior lip of the foramen magnum (Figure 1A, 1B). The tumor measured 37.1 mm oblique sagittal, 19.5 mm height, and 35.1 mm oblique coronal. The medulla was pushed posteriorly, and the cerebellar tonsils were compressed. The right vertebral artery was encased at the foramen magnum. There was no evidence of a hydrocephalus.

**Figure 2:**
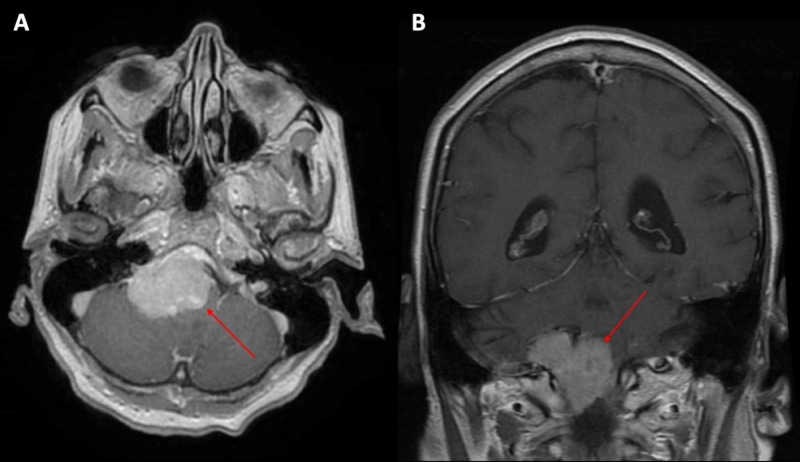
(A) Axial and (B) coronal views of magnetic resonance imaging of the brain at the time of treatment planning revealing right inferior clival meningioma.

Due to the complexity of the tumor given its location and impingement on essential structures (brainstem, cranial nerves) and high risk for development of obstructive hydrocephalus, many consultations were pursued in three different provinces driven by the medical teams. These included neurosurgical opinions for highly specialized radiotherapy techniques. In particular, there was an initial consultation to neurosurgery, referral to radiosurgery for Gamma Knife in another province, referral to another neurosurgeon for a second opinion, referral and work-up by a radiation oncology team that favored pursuing an out-of-province neurosurgery opinion prior to initiating external beam radiotherapy. All consultations suggested to pursue conventional external beam radiotherapy. The third neurosurgeon referred the patient to another out-of-province radiation oncology program, and her case was presented at the regional tumor board to expedite treatment. At the time of the planning MRI, the tumor measured 42.3 mm oblique sagittal, 21.1 mm height, and 38.8 mm oblique coronal (Figure 2A, 2B). External beam radiotherapy was started shortly thereafter. This process of obtaining multiple opinions lasted approximately 12 months.

By this time, she reported worsening occipital headaches. She also noticed decreased energy, generalized weakness in her limbs, and a significant decline in her balance in the previous three months. She tended to veer to the left when walking. This symptom was worse in open spaces and when walking down stairs. She also reported episodes of difficulty swallowing liquids in the left side of her throat.

## Discussion

According to the Canadian Institute for Health Information, the recommended maximum wait time between the time a specialist determines that a cancer patient is ready to receive radiation and start of radiation treatment is 28 days. In 2018, 97% of cancer patients in Canada received radiation treatment within this time frame [4]. The Canadian Association of Radiation Oncology has set the national benchmark of four weeks for radiation therapy (two weeks for consult and two weeks for treatment) [5]. In this case, the patient had a treatment interval of almost one year due to the multiple referrals and delays. This case highlights that although providing our patients with the best possible treatment option is important, timeliness and availability of treatment should also be prioritized. 

There was room for improvement in coordinating care between radiation oncology and neurosurgery. For example, after being seen for external beam radiation therapy the patient was referred again to surgery for a third opinion by provider request. However, prior to that referral she had already been assessed by neurosurgery and radiosurgery teams, who had recommended an external beam based treatment. The repeated referral-seeking delayed her eventual radiation therapy by another few months. Potential lack of clear communication between specialists could have played a role in this case.

The patient was not presented in a multidisciplinary tumor case conference until after consultation by the initial radiation oncologist, radiosurgery team, and the three neurosurgery teams. Case conferences are excellent opportunities to discuss challenging cases and determine care in a timely manner. Patients discussed in multidisciplinary team meetings are more likely to receive more accurate diagnosis and staging and have higher rates of treatment [6]. This case highlights the importance for regional tumor boards and case discussions to help expedite treatment, particularly when specific treatment modalities are only offered in certain centers. Communication of all teams that manage neuro-oncology tumors can continue to be improved.

The patient was also referred to specialists in three different provinces. Accessing three separate and distinct healthcare systems posed a barrier to information and management continuity for the patient. Currently, there is no national healthcare information system to share medical information such as lab reports, imaging, or consultation notes from other physicians. This would have made it more difficult for the different physicians to be able to communicate, collaborate, and share information with one another. This lack of continuity resulted in duplication of assessments, excess healthcare costs, and led to treatment delay. 

It is also important to recognize the significant costs to the patient for the multiple referrals in the various provinces. The patient had to physically travel to the appointments out of province which are associated with personal travel costs and time off work. Only one of the out-of-province referrals was delivered via telemedicine. As a result, virtual consultations could have played a larger role in this case. Telemedicine has the potential to reduce barriers to access of service as well as mitigate travel costs for patients in radiation oncology [7].

This case is also relevant to the concept of a medical home [8]. Having seen multiple specialists who referred the patient on to the next service, there was no primary care team responsible for centralizing the patient’s care as well as advocating for timely treatment. As a result, it would have been easy for the patient to get lost in the medical system. Short wait time ensures the best outcomes from radiation treatment and minimizes stress on the patient [9]. As radiation therapy does not tend to shrink meningiomas, but rather halt further growth, earlier treatment could have prevented the development of her decrease in balance, generalized weakness, and difficulty swallowing. 

## Conclusions

We report a case of a right inferior clival meningioma. Due to multiple referrals, treatment was delayed for over a year. Although eventually treated with radiation therapy, the delay of treatment led to significant progression of the patient’s symptoms, especially balance and cranial nerve dysfunction.
